# Supplementary material for: Removal of seed coat H3K27me3 in Arabidopsis requires noncanonical functions of brassinosteroid receptors
Source: Plant Cell. 2026 Jun 13;38(6):koag182. doi: 10.1093/plcell/koag182 (PMC13389951; doi:10.1093/plcell/koag182)
Supplement: koag182_Supplementary_Data [file koag182_supplementary_data.zip › Pankaj_Supplement.pdf]

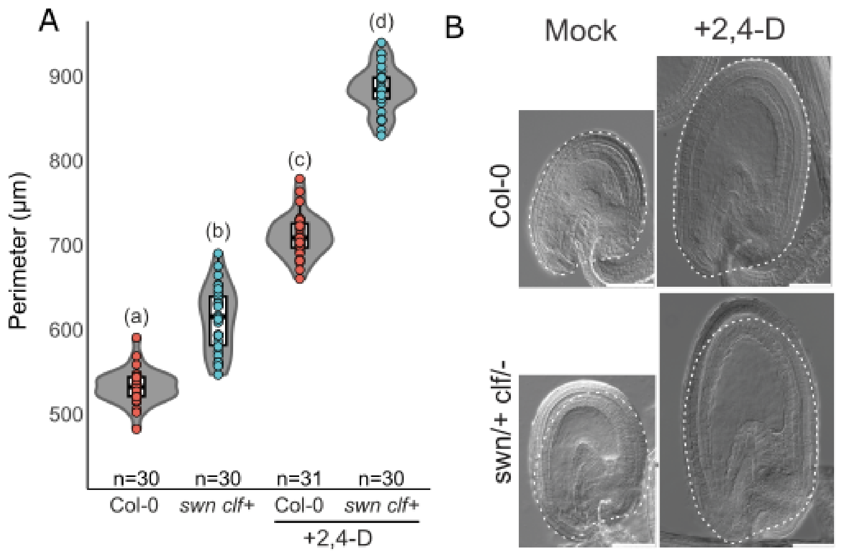

C

Name	Locus	Known Histone Targets	Expression in the Seed Coat
JMJ11/ELF6	AT5G04240	H3K27me2/3	
JMJ12/REF6	AT3G48430	H3K27me2/3; H3K36me2/3; H3K4me2/3	Not Determined
JMJ13	AT5G46910	H3K27me2/3	
JMJ14	AT4G20400	H3K4me1/2/3	
JMJ15/MEE27	AT2G34880	H3K4me3	
JMJ16	AT1G08620	H3K4me1/2/3	
JMJ17	AT1G63490	H3K4me1/2/3	
JMJ18	AT1G30810	H3K4me2/3	
JMJ19	AT2G38950	H3K4me3	
JMJ20	AT5G63080	H4R3me2	
JMJ21	AT1G78280	Not Determined	
JMJ22	AT5G06550	H4R3me2	
JMJ24	AT1G09060	H3K9me2	
JMJ25/IBM1	AT3G07610	H3K9me1/2	
JMJ26	AT1G11950	Not Determined	
JMJ27	AT4G00990	H3K9me1/2	
JMJ28	AT4G21430	H3K9me1/2	
JMJ29	AT1G62310	H3K9me1/2; H3K4me3	
JMJ30	AT3G20810	H3K27me2/3; H3K36me2/3	
JMJ31	AT5G19840	Not Determined	
JMJ32	AT3G45880	H3K27me2/3	

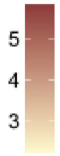

Figure S1, supports Fig. 1. (**A**) Seed size differences of auxin-induced autonomous seeds in WT and *swn clf*/+ at 3 days after auxin treatments (3 DAT). The letters indicate statistical significance for p-value <0.05 (ANOVA). Representative photos are seen in (**B**). Scale bars indicate 50 µm. (**C**) List of known JMJ-type demethylases with their putative substrates and expression levels in seed coats of seeds at the pre-globular embryo stage. The bar on the right-hand side represents the relative expression values, as previously determined (Belmonte et al., 2013).

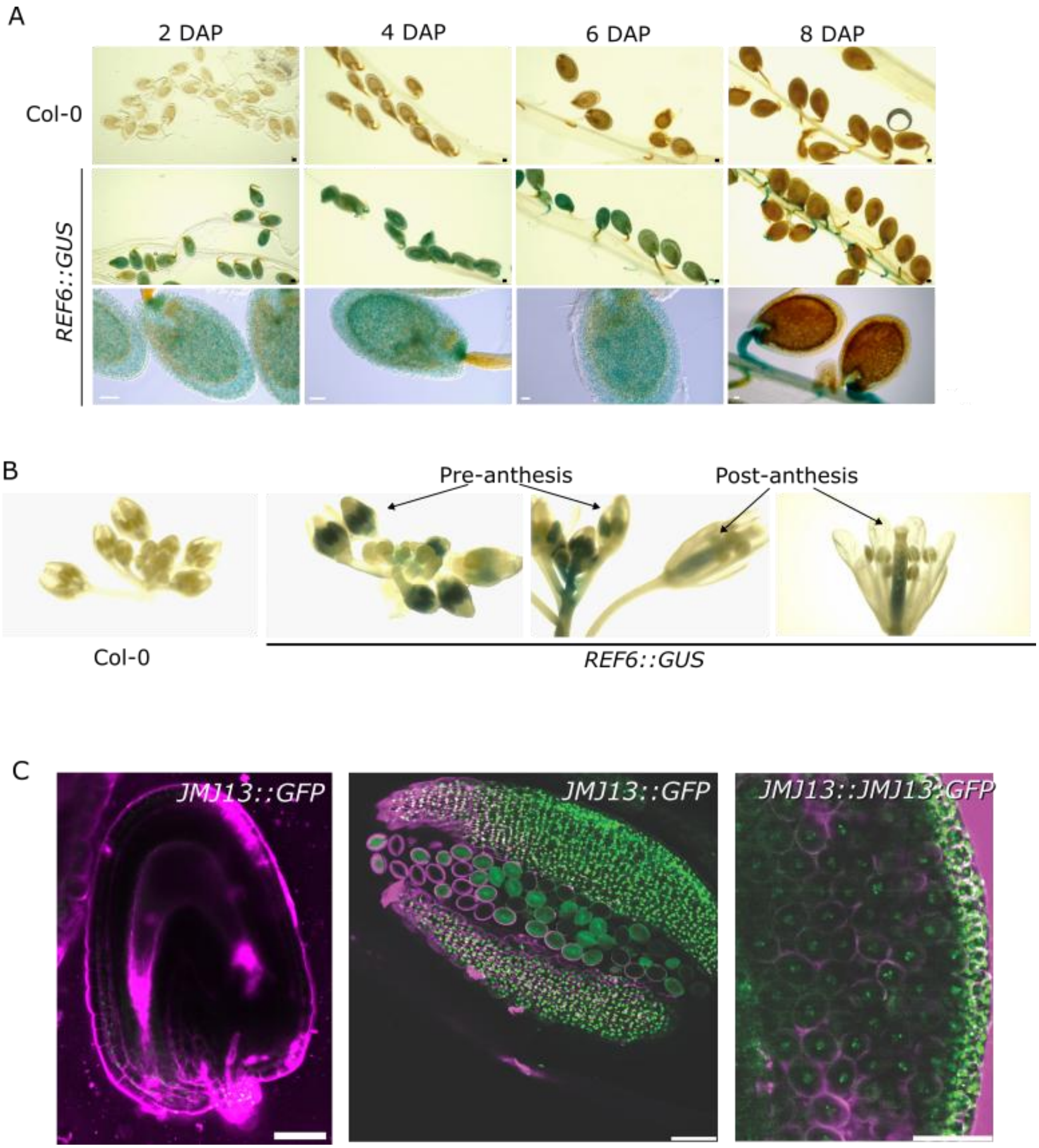

Figure S2, supports Fig. 1. Tissue specific expression of JMJ demethylases. (**A-B**) Expression of REF6 as determined by beta-glucuronidase activity in developing seeds (**A**) and flowers (**B**). Untransformed plants (Col-0) were used as negative control. (**C**) Expression of *JMJ13* in seeds and anthers as determined by *JMJ13::GFP and JMJ13:JMJ13:GFP*. Scale bars indicate 50 µm.

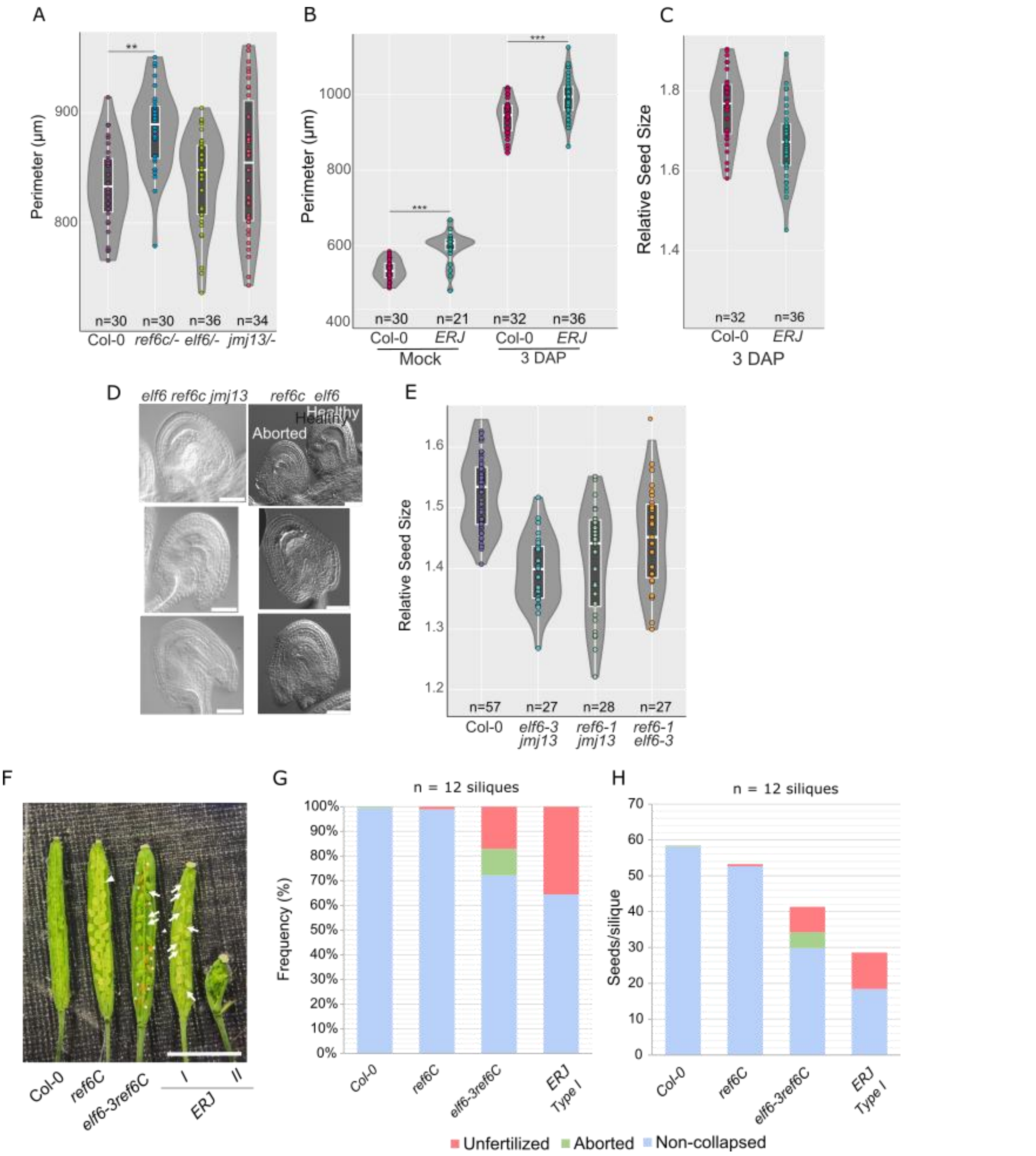

Fig. S3, supports Fig. 1. (**A**) Size of fertilized seeds (3 DAP) for the single *jmj* mutants and respective WT. (**B**) Size of unfertilized ovules (Mock) and sexual seeds (3 DAP) of the *ERJ* mutant and respective WT. Differences are significant for p<0.01 (*), p<0.001 (**) or p<0.00001 (***) (ANOVA). (**C**) Autonomous and sexual seed size of *elf6 ref6c jmj13 (ERJ)* and the respective WT. The relative seed size was calculated as a ratio between the perimeter of each seed and the average perimeter of unfertilized ovules of their respective genotype. (**D**) Aborted ovules of *ERJ* (left) and *ref6c elf6* (right). Scale bars indicate 50 µm. (**E**)

Relative size of WT and *jmj* double mutant seeds at 3 DAP. (**F**) Morphology of *ref6c*, *elf6 ref6c and ERJ* siliques. Two types of siliques are obtained in *ERJ:* Type I siliques are more morphologically similar to the WT, while Type II siliques are malformed and often show “carpel-within-carpel” phenotypes (more details in **Fig. S4**). Aborted seeds are indicated by arrows and unfertilized/underdeveloped ovules are indicated with asterisks. Scale bar indicates 5 mm. (**G**) Quantification of seed phenotypes in Col-0, *ref6C*, *elf6-3 ref6C* and *ERJ*, scored as indicated in (**F**). (**H**) Mature seed set of Col-0, *ref6C*, *elf6-3 ref6C* and *ERJ*. Note the reduced seed set of ERJ. For (**G**) and (**H**) only Type I siliques were taken into account. The seeds from twelve independent siliques were counted: three siliques of four different plants each.

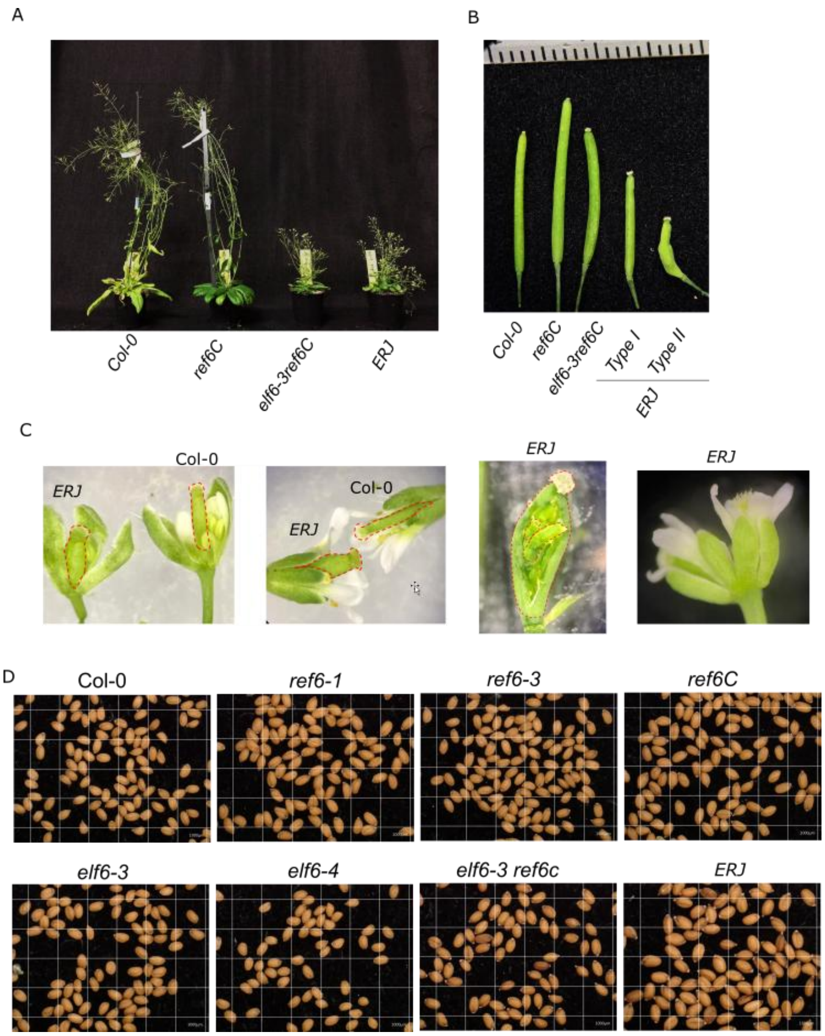

Fig. S4, supports Fig. 2. **Vegetative and seed phenotypes of Col-0 and *jmj* mutants.** (**A**) Overall plant morphology of Col-0, *ref6c*, *elf6-3 ref6c* and *ERJ*. Note the dwarfism of *efl6-3 ref6c* and *ERJ*. (**B**) Bent silique phenotype observed in Type II siliques of *ERJ* (also described in **Fig. S3**). (**C**) Floral phenotypes of *ERJ*, showing swollen pistils and pitil-within-pistil phenotypes. None of these phenotypes are observable in the other genotypes, including in *elf6-3 ref6c*. (**D**) Mature seed phenotypes of Col-0, *ref6* and *elf6* single mutants and higher order *elf6-3 ref6c* and *ERJ* mutants.

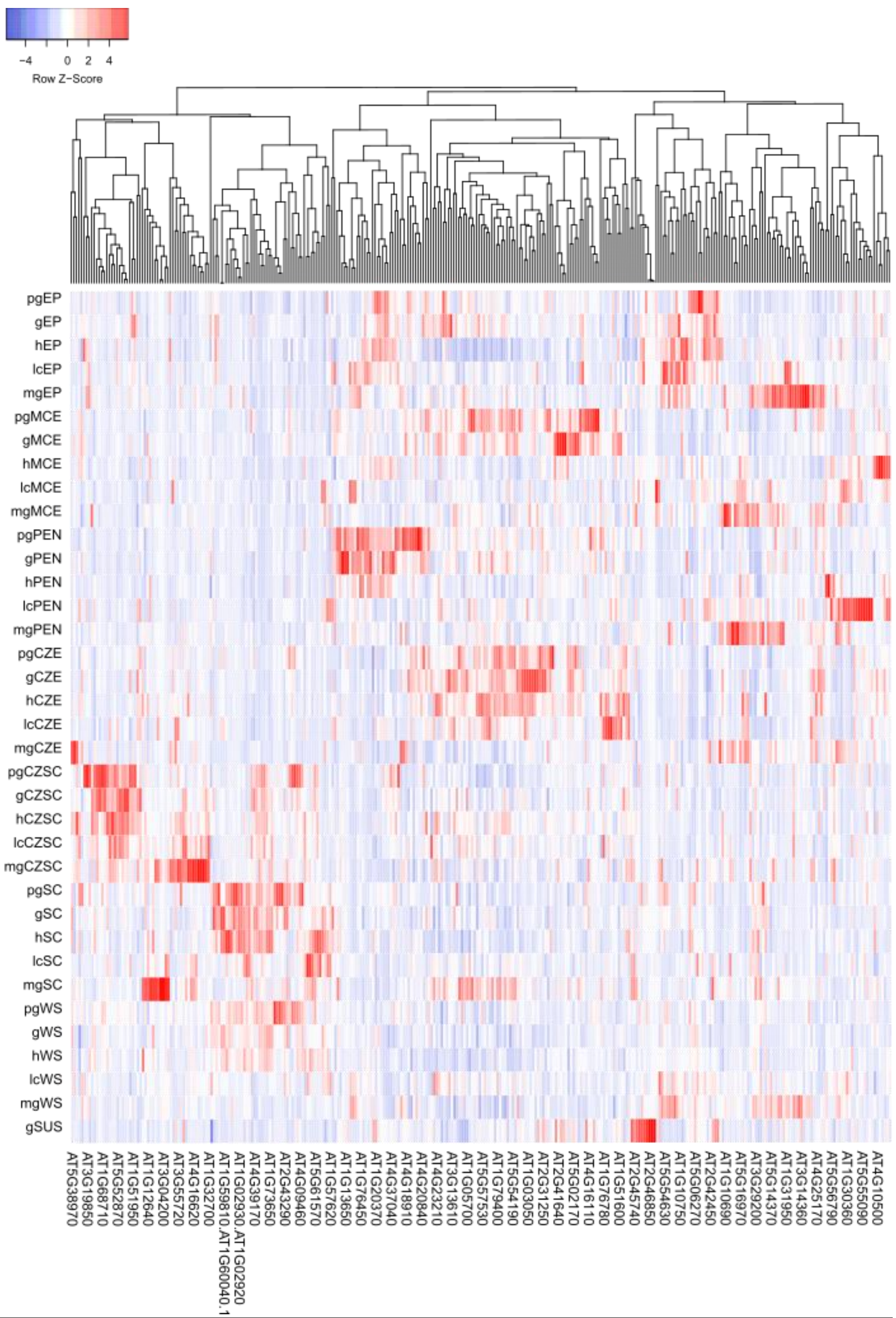

Fig. S5, supports Fig. 2. Relative expression of genes carrying REF6 binding sites, specifically expressed in the embryo proper (EP), micropylar endosperm (MCE), peripheral endosperm (PEN), chalazal endosperm (CZE), chalazal seed coat (CZSC), seed coat (SC), whole seed (WS) and suspensor (SUS). Gene expression data is based on (Belmonte et al., 2013). The seed stages indicated are: pg, pre-globular; g, globular; h, heart; lc, linear cotyledon; and mg, mature green. Only genes with at least 4 REF6 binding sites were included in the analysis.

A

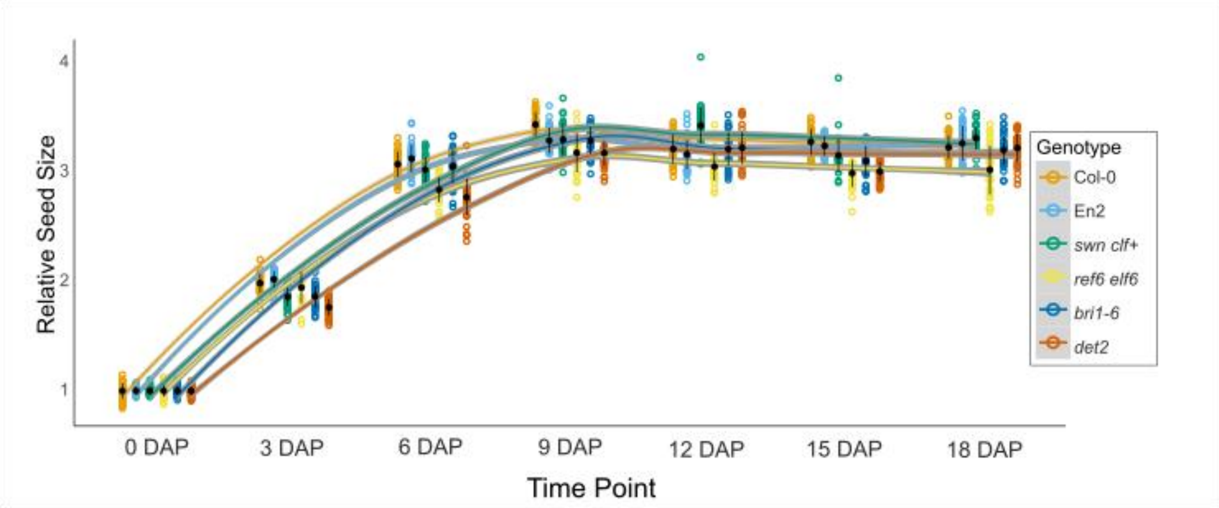

B

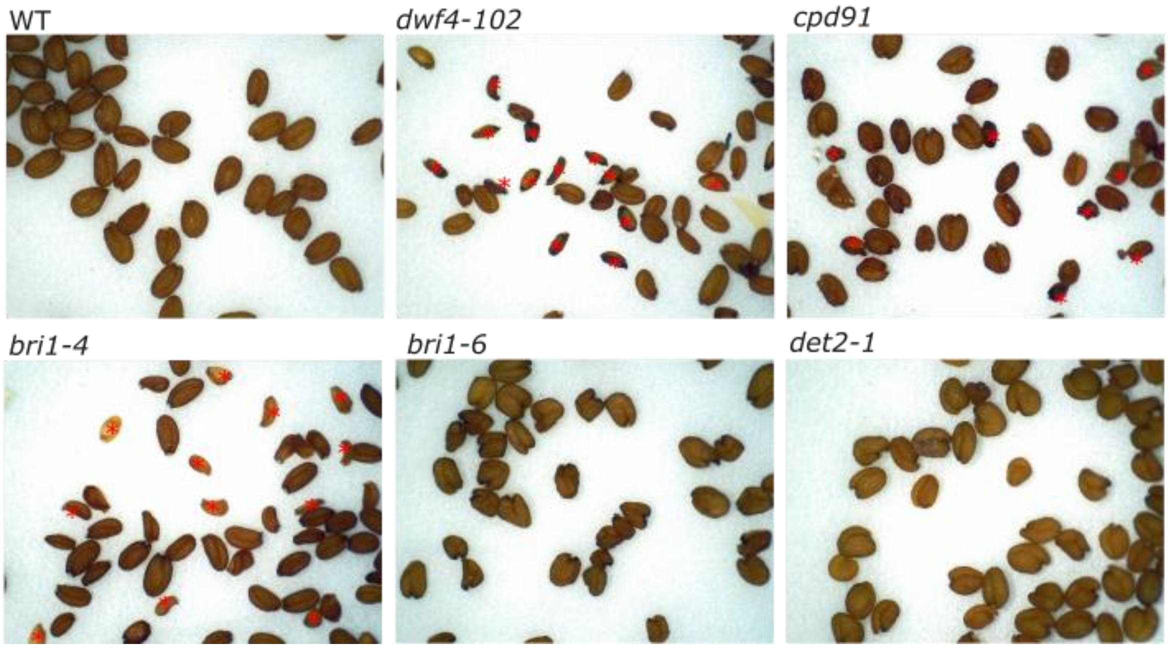

Fig. S6, **supports Fig. 3**. (**A**) Seed growth kinetics of the main genotypes used in this manuscript from 0 to 18 DAP. Seed size is normalized to the size of mature ovules of each genotype at 0 DAP. (**B**) Mature seed phenotypes of WT, strong (*dwf4-102*, *cpd91*, *bri1-4*) and weak (*bri1-6*, *det2-1*) BR mutants. Red asterisks indicate aborted or severely malformed seeds in the strong mutants. The weak mutants produce full sets of viable seeds.

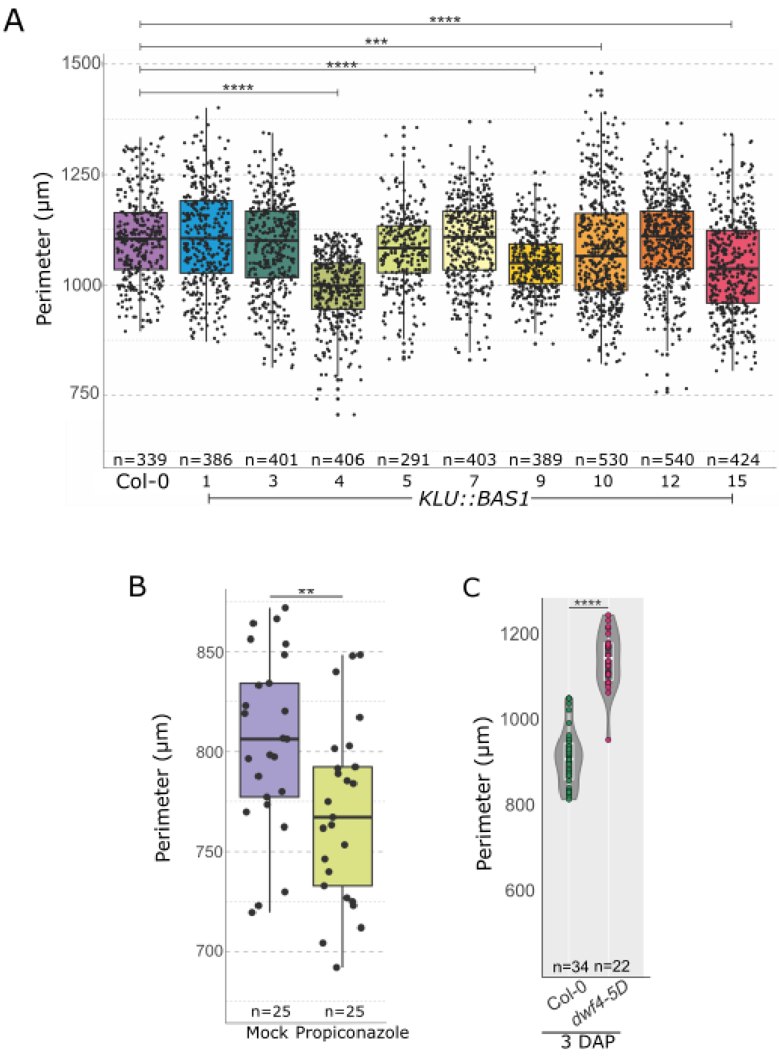

Fig. S7, supports Fig. 3. (**A**) Seed perimeter of dry seeds of transgenic lines expressing *KLUH::BAS1*. (**B**) Seed size at 3 DAP after treatments with the BR inhibitor propiconazole. (**C**) Perimeter of 3 DAP seeds of the BR overproducing mutants *dwf4-5d*. Asterisks indicate statistical significance for p-value <0.01 (**), <0.001 (***), and <0.0001 (****), as determined by Anova.

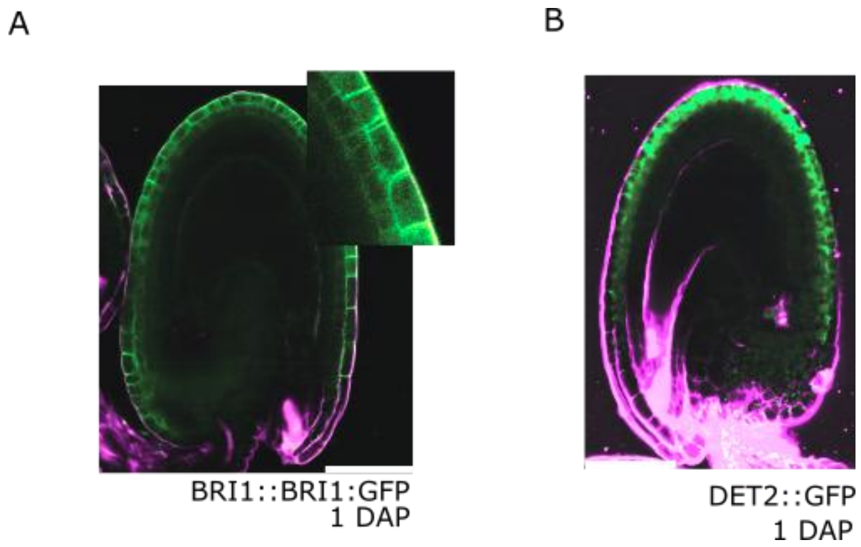

Fig. S8, supports Fig. 4. Expression of *BRI1::BRI1:GFP* (**A**) and of *DET2::GFP* (**B**) in seeds at 1 DAP. Magenta is propidium iodide (PI). Scale bars indicate 50 µm.

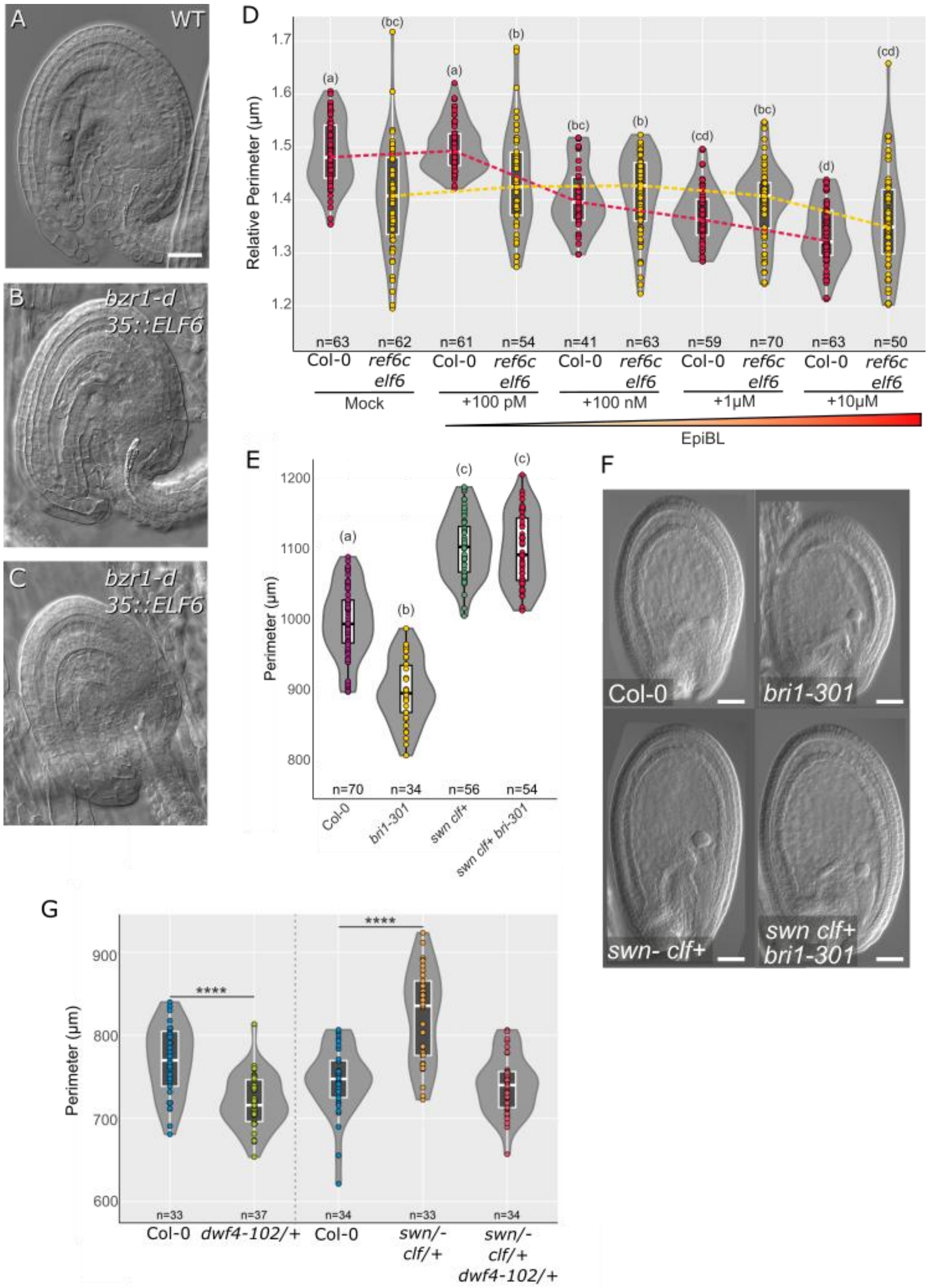

Fig. S9, supports Fig. 5. (**A-C**) Examples of WT and of malformed ovules obtained in lines expressing *CaMV35S::ELF6 bzr1-D*. No such phenotypes were observed in the single mutants. Scale bars indicate 50 µm. (**D**) Perimeter of auxin+epi-BL treated WT (Col-0) and *ref6 elf6* seeds at 3 DAT. The letters indicate statistical significance for p-value <0.05 (ANOVA). (**E**) Perimeter of seeds of an independent *bri1-301* allele and its interactions with PRC2 mutants. The letters indicate statistical significance for p-value <0.05 (Anova). Examples of seeds can be seen in panel (**F**). The scale bar indicates 50 µm. (**G**) Perimeter of seeds after auxin application in WT, *swn clf*/+, *dwf4-102* and respective triple mutant. **** indicates p-value <0.0001 (ANOVA).

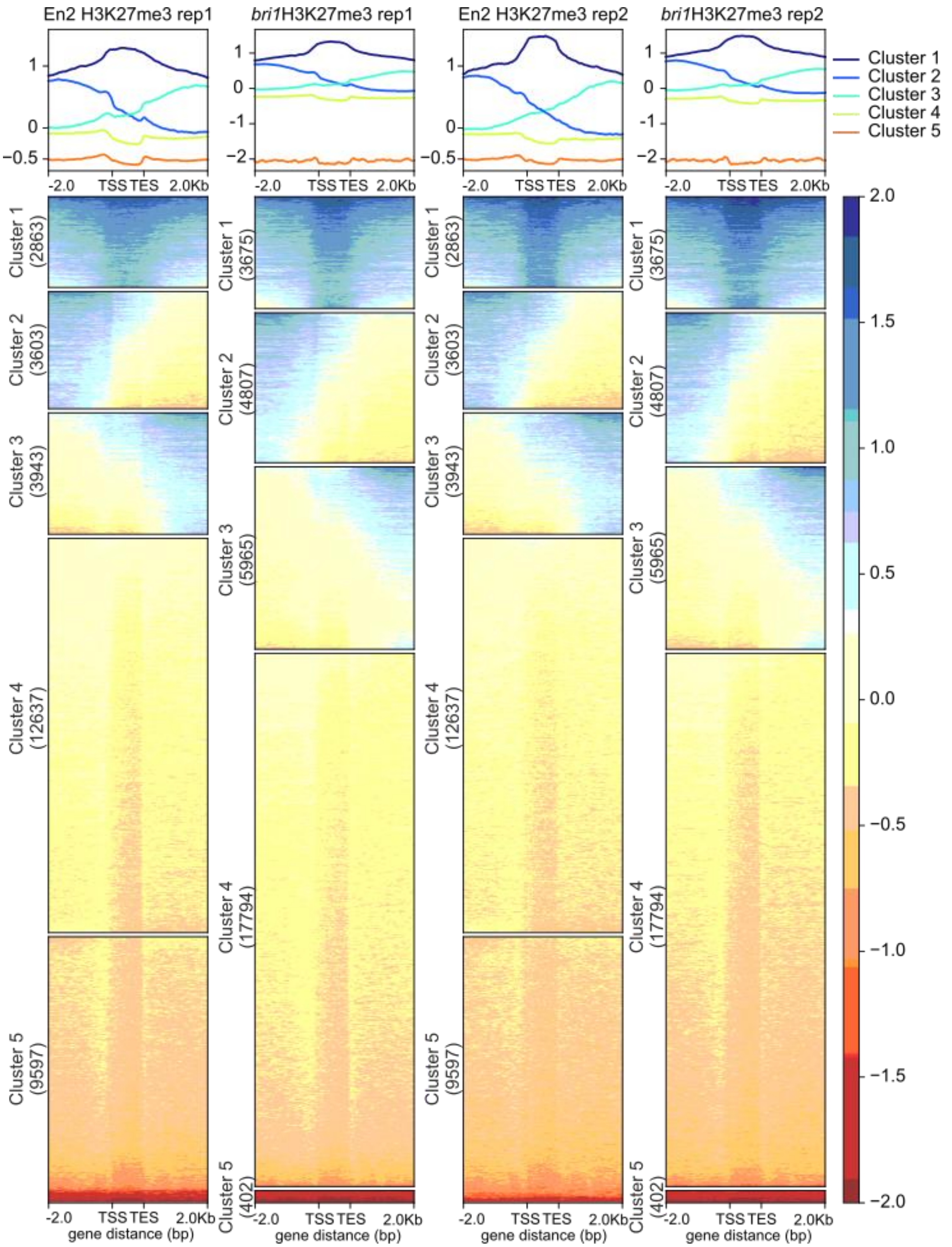

Fig. S10, supports Fig. 6. Clustering analysis of H3K27me3 profiles in WT En-2 and in *bri1*. The two replicates are shown for each genotype. The metagene plots on top indicate the average enrichment of H3K27me3 over gene regions for each of the Clusters indicated below. The colors indicate relative enrichment. TSS - transcription start site, TES - transcription end site.

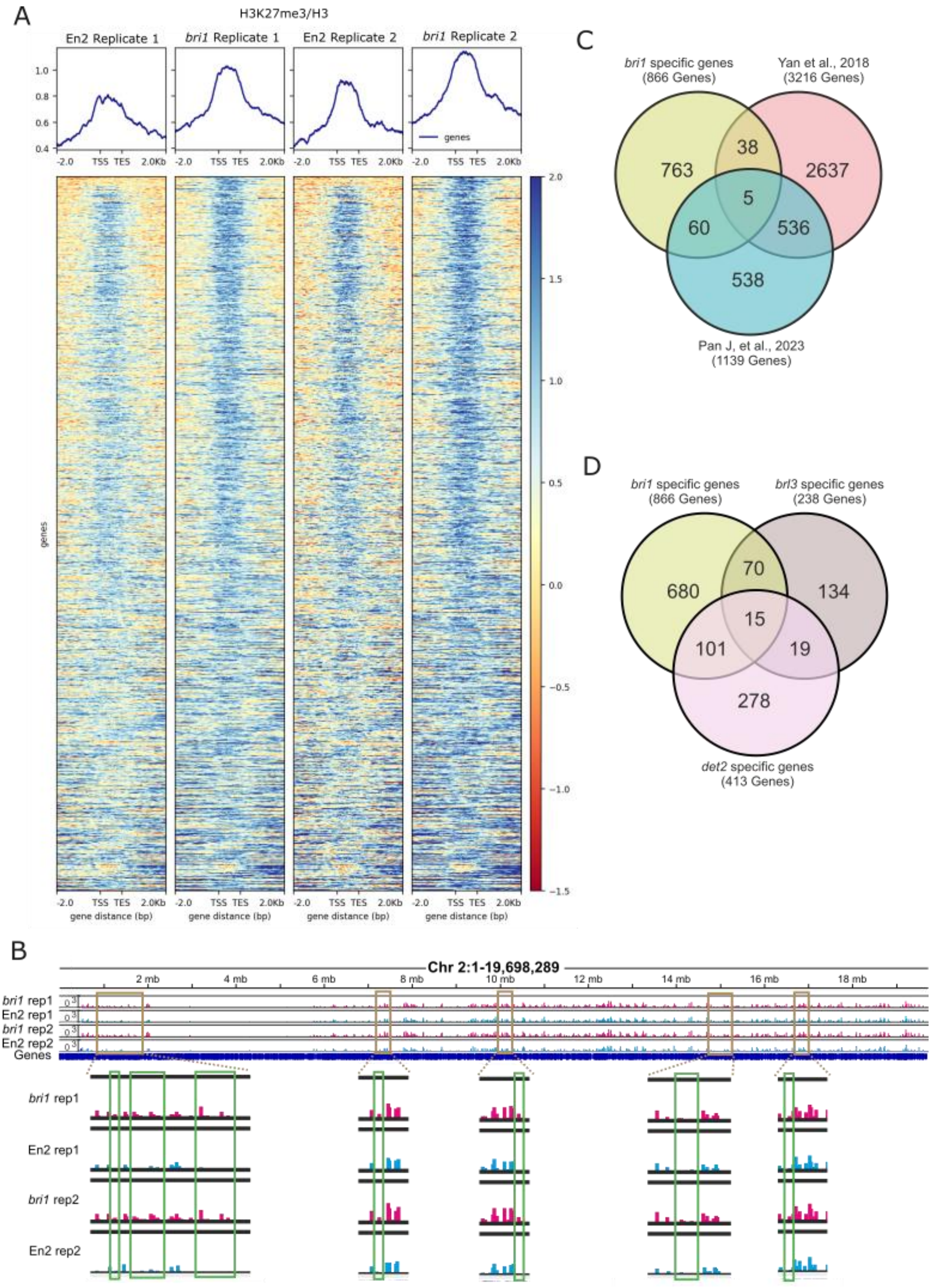

A
H3K27me3/H3
En2 Replicate 1
bri1 Replicate 1
En2 Replicate 2
bri1 Replicate 2
genes
gene distance (bp)
TSS TES
-2.0
2.0Kb
B
Chr 2:1-19,698,289
bri1 rep1
En2 rep1
bri1 rep2
En2 rep2
Genes
C
bri1 specific genes (866 Genes)
Yan et al., 2018 (3216 Genes)
Pan J, et al., 2023 (1139 Genes)
763
38
2637
5
60
536
538
D
bri1 specific genes (866 Genes)
brl3 specific genes (238 Genes)
det2 specific genes (413 Genes)
680
70
134
15
101
19
278

Fig. S11, supports Fig. 6. (**A**) Ratios of H3K27me3 to H3 of all 867 *bri1*-enriched genes, in En-2 (WT) and *bri1* mutant. Two replicates are shown. TSS - transcriptional start site, TES - transcriptional end site. (**B**) H3K27me3/H3 profiles along Chromosome 2, illustrating H3K27me3 hypermethylation in some domains in *bri1* (green zoomed-in boxes), when compared to the respective En-2 WT. The global profiles remain mostly identical. Two biological replicates per genotype are shown. (**C**) Overlap between *bri1*-enriched genes and genes ectopically methylated in *ref6* and *ERJ* mutants, as previously published (Yan *et al.*, 2018; Pan *et al.*, 2023). (**D**) Overlap between genes with H3K27me3/H3 enrichment in *bri1*, *brl3* and *det2*.

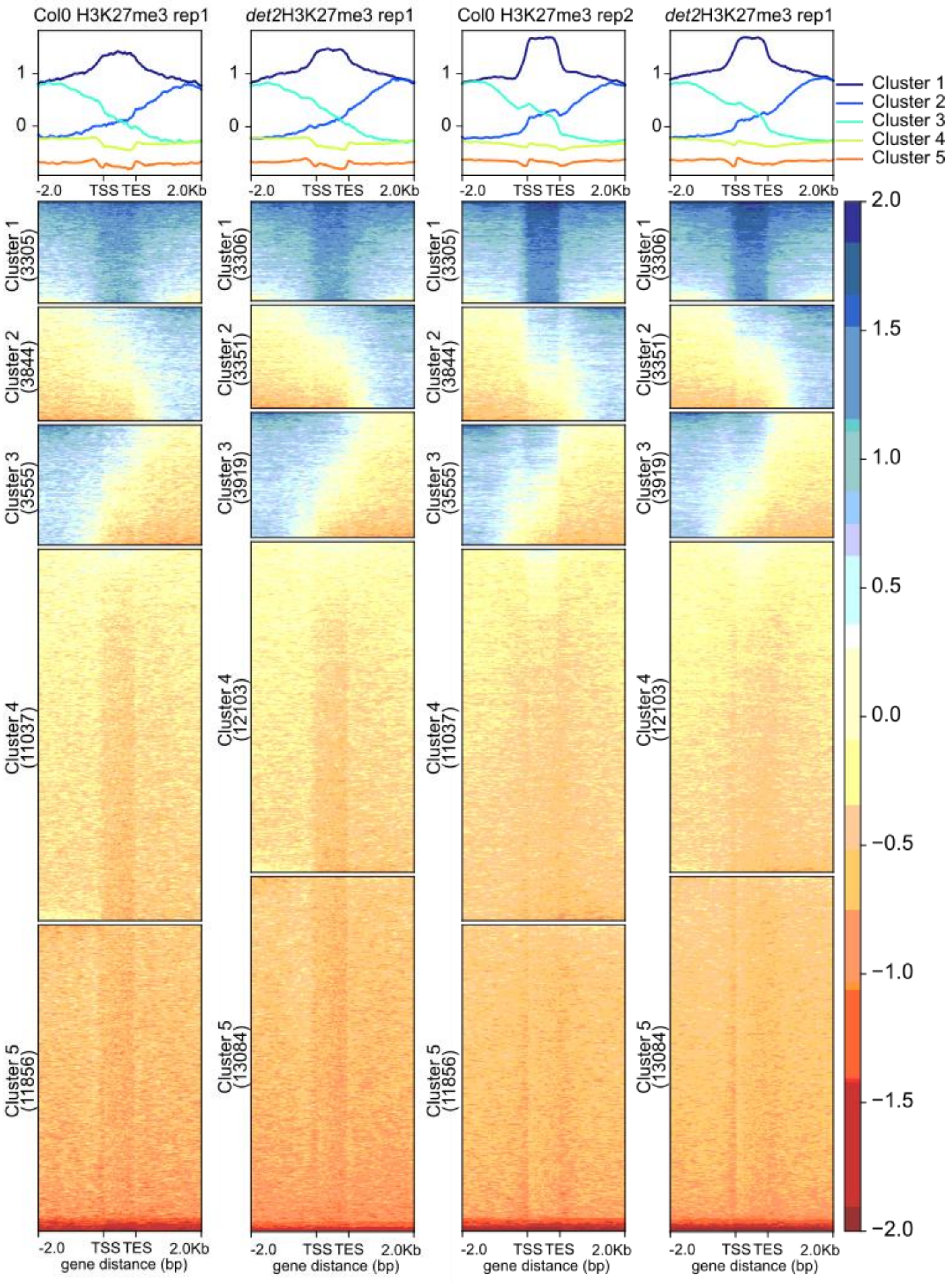

Fig. S12, supports Fig. 7. Clustering analysis of H3K27me3 profiles in WT Col-0 and in *det2*. The two replicates are shown for each genotype. The metagene plots on top indicate the average enrichment of H3K27me3 over gene regions for each of the Clusters indicated below. The colors indicate relative enrichment. TSS - transcription start site, TES - transcription end site.

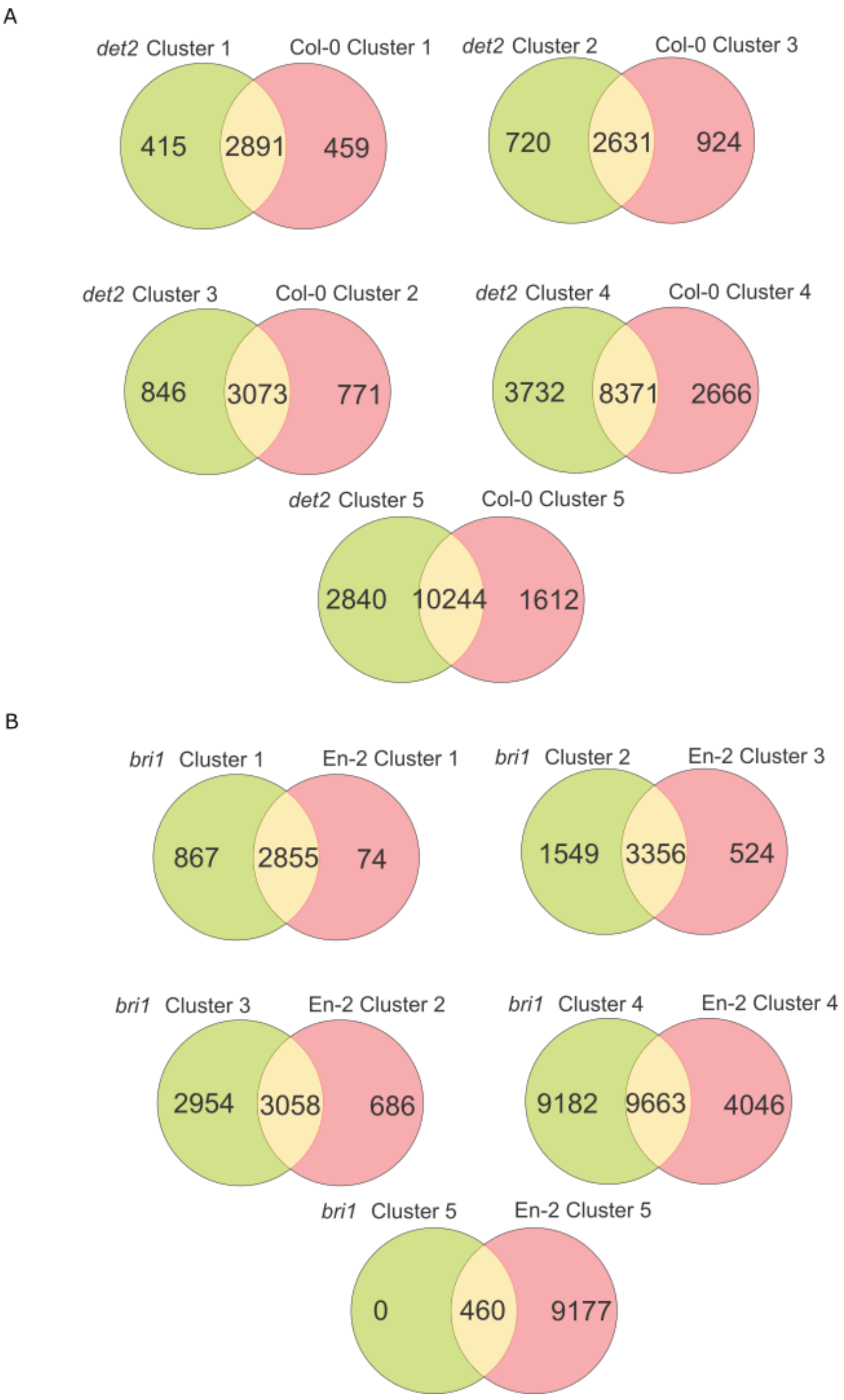

Fig. S13, supports Fig. 6 and 7. Overlaps between the number of genes labeled with H3K27me3 between *det2* and Col-0 (**A**) and *bri1* and En-2 (**B**) Clusters. Similar clusters were compared between the pairs of genotypes. Note that WT Clusters 2 were compared to the mutant Clusters 3. This is because WT Clusters 2 represent genes labeled in the promoter region, while genes labeled in the terminator are in Cluster 3. In both mutants this is the opposite.Therefore we decided to compare the most similar clusters between the genotypes.

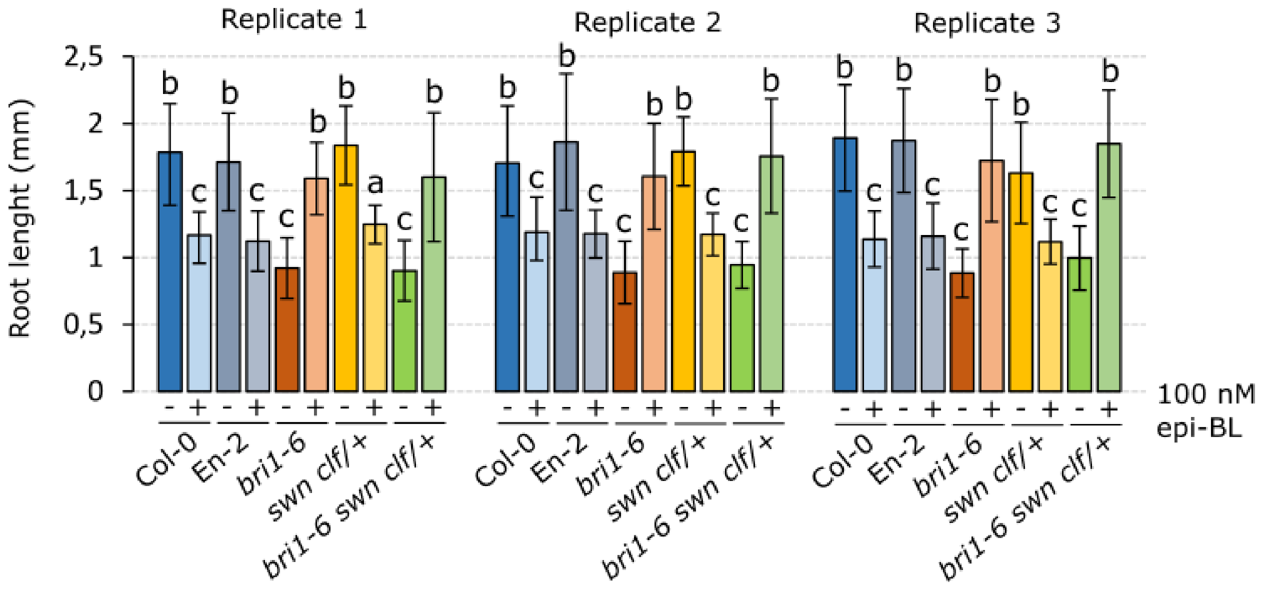

Fig. S14, supports Fig. 6 and 7. BR root sensitivity assay. Three independent replicates are shown, grown either in ½ MS plates or ½ MS supplemented with 100 nM epi-BL. The roots were measured at 7 days after germination. The bars indicate standard deviation. Letters indicate statistical significance for p-value<0.05 (ANOVA).

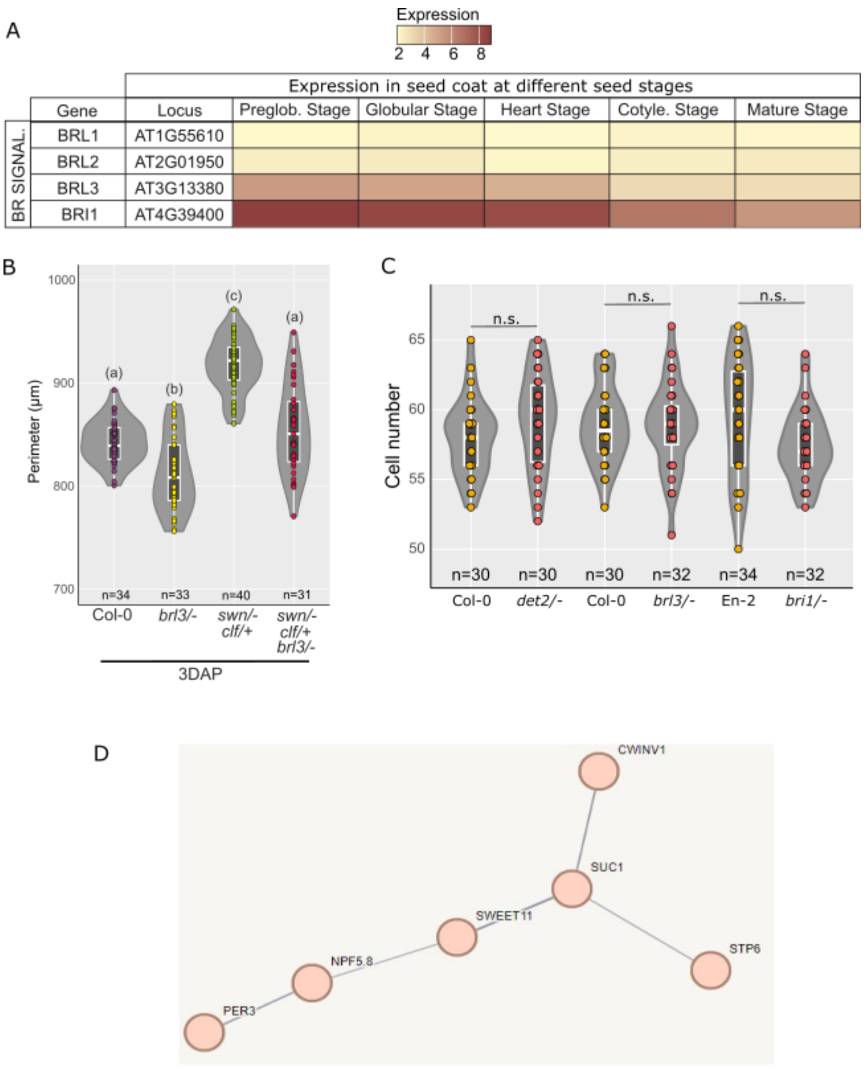

Fig. S15, supports Fig. 7. (**A**) Expression of *BRL* genes in different stages of seed development. (**B**) Seed perimeter of Col-0, *brl3*, *swn clf*/+, and respective triple mutant, at 3 DAP. The letters indicate statistical significance for p-value <0.05 (ANOVA). The numbers at the bottom indicate the number of seeds analysed. (**C**) Number of cells in the outer cell layer of the seed coats of the indicated genotypes at 3 DAP. The letters indicate statistical significance for p-value <0.05 (ANOVA). The numbers at the bottom indicate the number of seeds analysed. (**D**) Cluster of sugar transport related genes differentially methylated by H3K27me3 in *brl3* mutants.

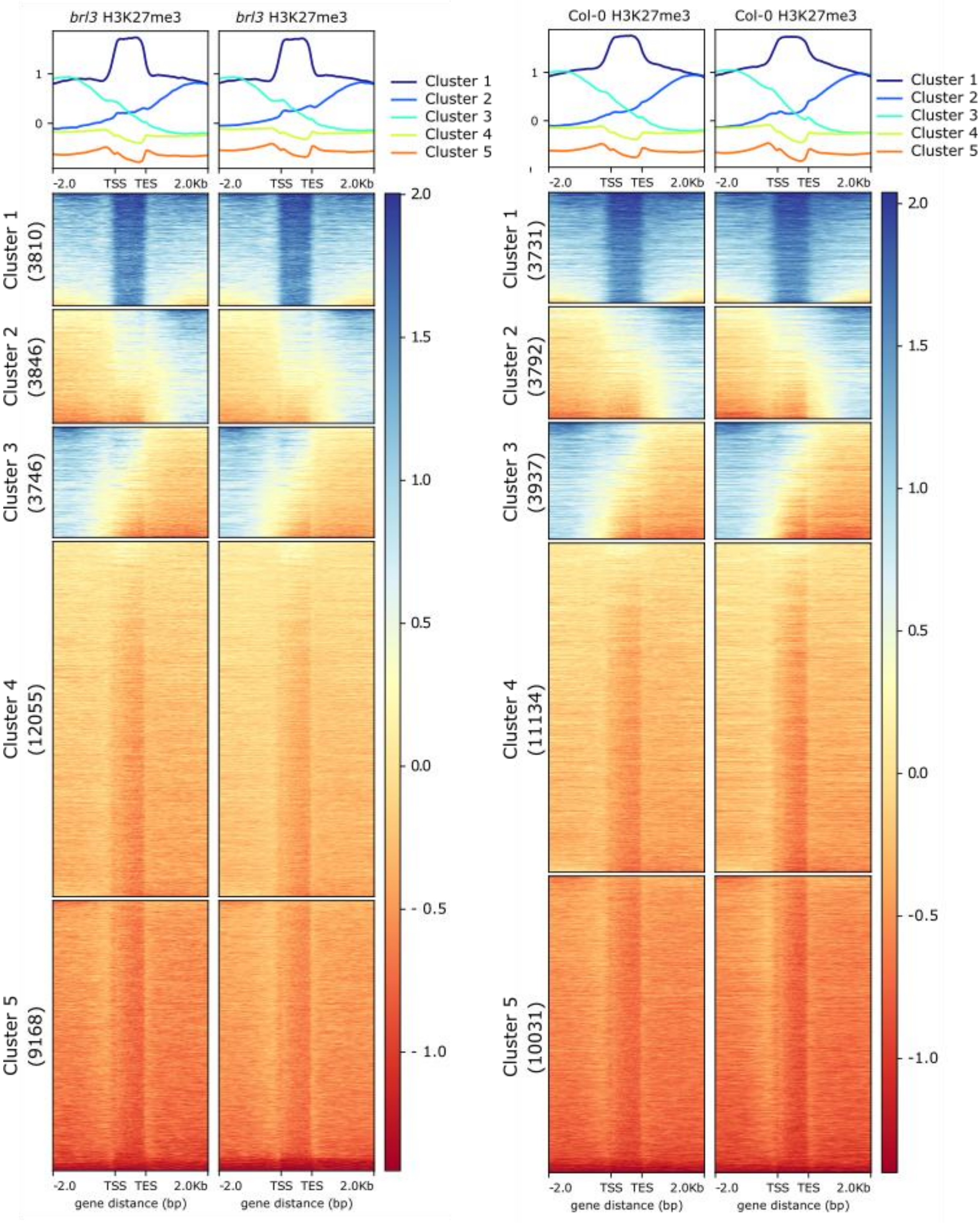

Fig. S16, supports Fig. 7. Clustering analysis of H3K27me3 profiles in WT Col-0 and in *brl3*. The two replicates are shown for each genotype. The metagene plots on top indicate the average enrichment of H3K27me3 over gene regions for each of the Clusters indicated below. The colors indicate relative enrichment. TSS - transcription start site, TES - transcription end site.

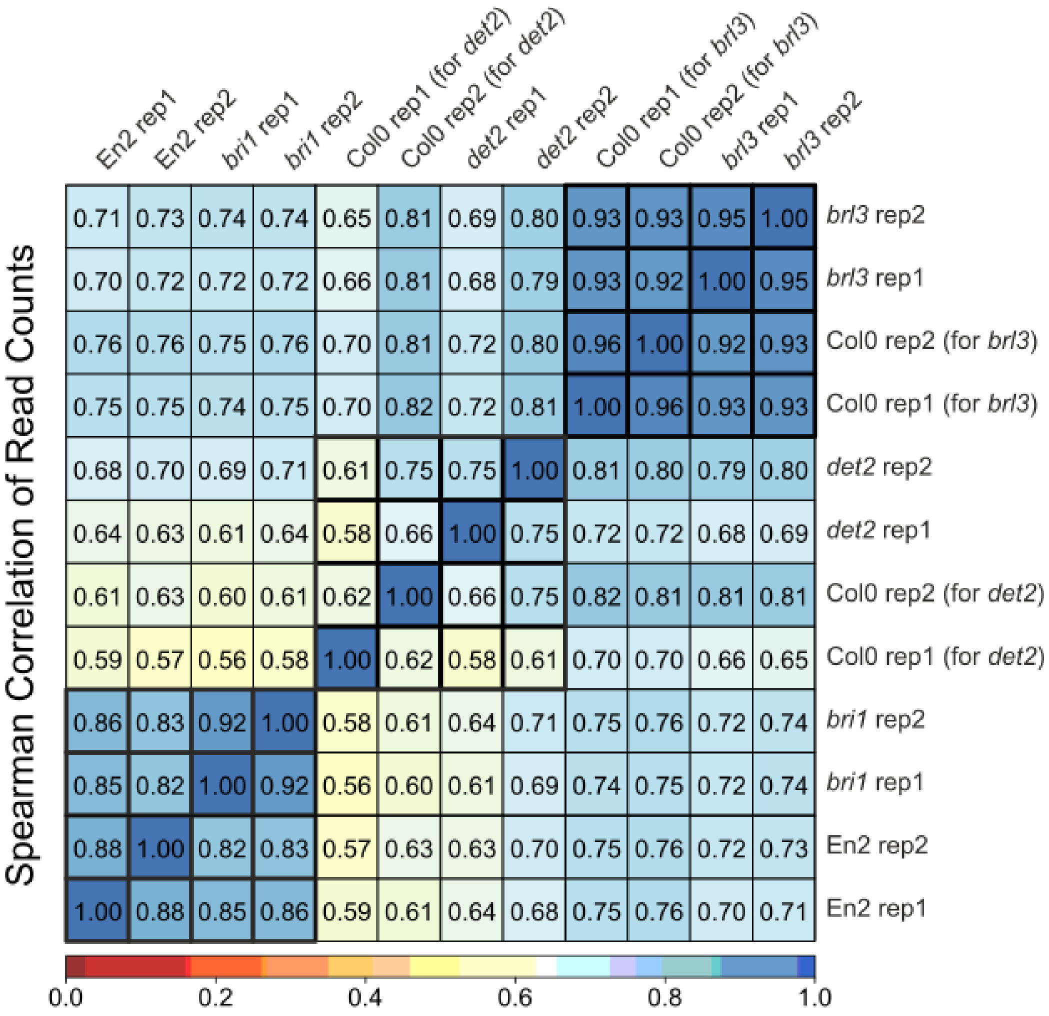

Fig. S17, supports Fig. 6 and 7. Spearman correlations for read counts, as obtained for H3K27me domain regions for the CUT&TAG analyses.

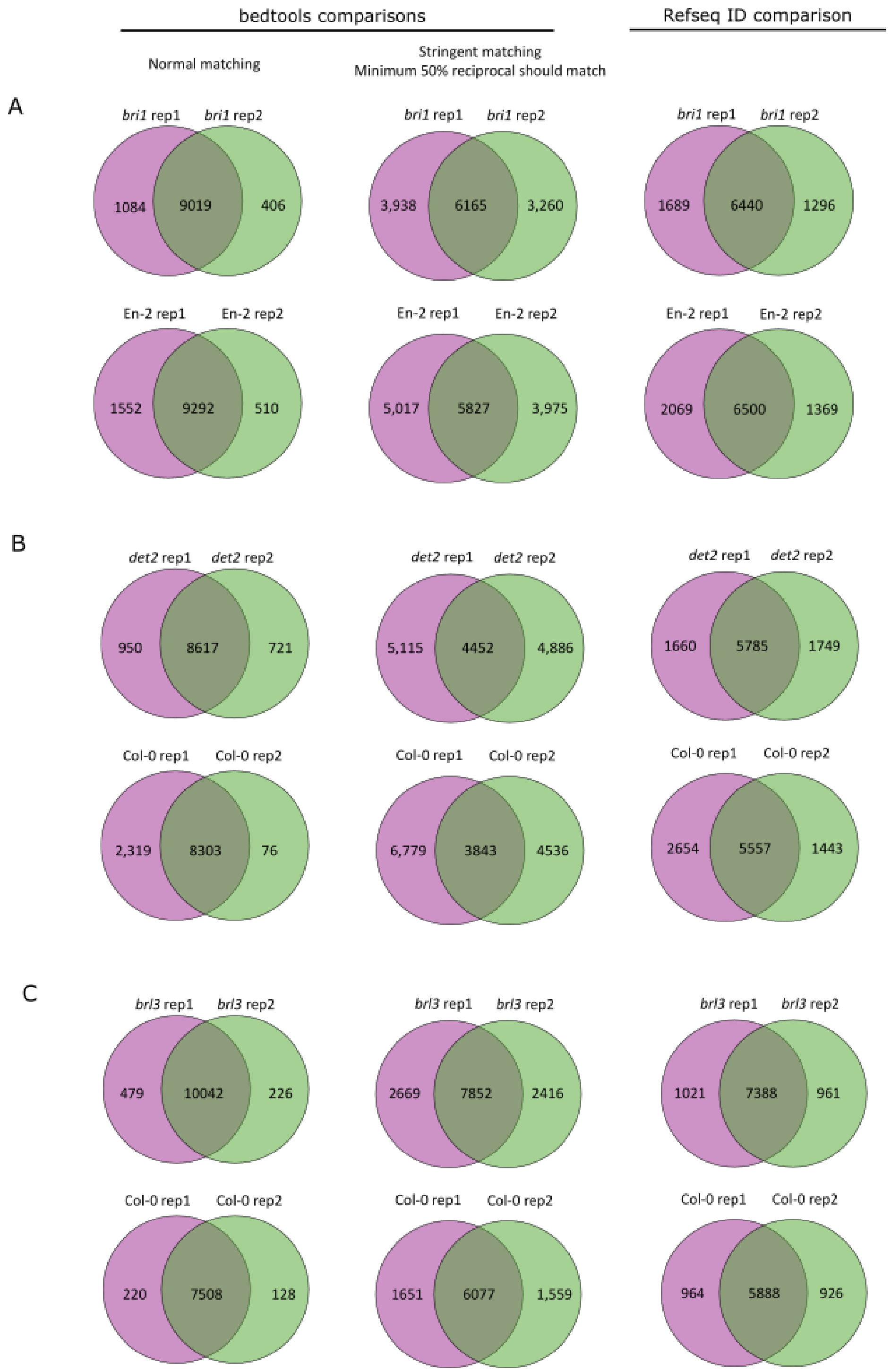

Fig. S18, supports Fig. 6 and 7. Overlaps of CUT&TAG peaks. Overlaps for all biological replicates of CUT&TAG peaks as determined by bedtools (**A-B**) and Intervene using RefSeq IDs (**C**). For bedtools both normal (any % match) and stringent (minimum 50% peak match) were done. (**A**) En-2 vs *bri1*. (**B**) Col-0 vs *det2*. (**C**) Col-0 vs *brl3*.

Table S1: Primers used for genotyping and cloning.

Primer	Genotype	Sequence	Information
p2_1	*jmj13*	TCAAGTTCCATTTGCTGCTTC	WT p2_1 & p2_2
p2_2	*jmj13*	GGAATGTTTGCTGACACTTGG	Mut p2_2 &p2_3
p2_3	*jmj13 tdna (GABIKAT)*	TGGTTCACGTAGTGGGCCATCG	
p2_4	*elf6-3*	ACGTCAATGCGGTAATCATTC	WT p2_4 & p2_5
p2_5	*elf6-3*	TTTGCAGATCCCATTGCTTAC	Mut p2_5 & p2_8
p2_6	*ref6-1*	TCATATACAAGGCGTTCGGTC	WT p2_6 & p2_7
p2_7	*ref6-1*	CAGTTGCAACTCTGGAGAAGG	Mut p2_7 & p2_8
p2_8	*SALK T-DNA LBb1.3*	ATTTTGCCGATTTCGGAAC	genotyping SALK lines
p2_9	*ref6c*	GGTTGCTCCAGAGTTCAGACC	Mutant band smaller than WT
p2_10	*ref6c*	CATGGTCCTCCACATGCCAAGC	Mutant band smaller than WT
p2_11	*JMJ13 promoter gateway FP*	GGGGACAAGTTTGTACAAAAAAGCAGGCTTCttaaaaagggaataggagatttaag	cloning JMJ13 transcriptional reporter
p2_12	*JMJ13 promoter gateway RP*	GGGGACCACTTTGTACAAGAAAGCTGGGTTATCATTCCGTAATCTATTATCTCTG	cloning JMJ13 transcriptional reporter
p2_13	*JMJ13 promoter sequencing*	cccaagattttaagcaaagta	sequencing JMJ13 transcriptional reporter
p3_15	*dwf4-102*	GGCAGCTCCTACGTCATTAAAG	WT p3_15+p3_16
p3_16	*dwf4-102*	CCGGACATGAGACTTCTTCTG	Mut p3_16+p3_1
p3_17	*dwf4-5D* T-DNA	aaaggctatcgttcaagatgcc	
p3_18	*dwf4-5D*	AGCGTGTAACCATCTGCAAC	WT p3_18+p3_19
p3_19	*dwf4-5D*	GTCAAACCCGTAGACTTTTGGC	Mu p3_17+p3_19
p3_20	*bzr-1D*	CTAACTGGGAATCTATCGC	To be cut by HpaII
p3_21	*bzr-1D*	TCAACCACGAGCCTTCC	WT (127bp + 310 bp) Mu (437 bp)
p3_22	*brl3*	CCAGTGAACTCGTTTGAGCTC	WT p3_22+p3_23
p3_23	*brl3*	TTTATCGAACACTTTGTGGGC	Mut p3_23+p3_1
p3_24	*swn-3*	CGTTTCCGAGGATGTCATTGTG	WT p3_24+p3_25
p3_25	*swn-3*	TGGAACTTTTGAGTGGCTAGAGGTG	Mu p3_25+p3_26

p3_26	SALK T-DNA LBb1.1	GCGTGGACCGCTTGCTGCAACT	genotyping SALK lines
p3_27	*clf9*	tgggttcgtttaggaaccatt	WT p3_27+p3_28
p3_28	*clf9*	ccagcataacagttgacatagca	Mu p3_26+p3_28
p3_29	DET2 CDS	GGGGACAAGTTTGTACAAAAAAGCAGGCTatggaagaaatcgccgataaaac	cloning DET2 in rescue lines
p3_30	DET2 CDS	GGGGACCACTTTGTACAAGAAAGCTGGGTtcagtacacaaaaggaataacagct	cloning DET2 in rescue lines
p3_31	DET2 seq	TGAGCGAAACCCTATAAGAACCCT	Genotyping DET2 rescue lines p3_29+p3_31
p3_32	BRI1 CDS	GGGGACAAGTTTGTACAAAAAAGCAGGCTATGAAGACTTTTTCAAGCTTCTTTCT	cloning BRI1 in rescue lines
p3_33	BRI1 CDS	GGGGACCACTTTGTACAAGAAAGCTGGGTTCATAATTTTCCTTCAGGAACTTCT	cloning BRI1 in rescue lines
p3_34	BRI1 seq	GTGGGTTTGGAGATGTTTAC	Sequencing BRI1 in rescue lines
p3_35	BRI1 seq	GGTAAACGGCCAACGGATT	Genotyping BRI1 rescue lines p3_33+p3_35
p3_36	GUSplus CDS	gcgggatatcactagATGGTAGATCTGAGGGTAAATTTCT	Cloning GUSplus in pB7WG
p3_37	GUSplus CDS	ttgaacgatcctgcaTCAGTTCTTGTAGCCGAAATCTGGA	Cloning GUSplus in pB7WG
p3_38	REF6 promoter	ataattcgagggtacATGAAGAGTTGAGTGATGACACG	Cloning REF6 promoter in reporter lines
p3_39	REF6 promoter	cgtcgggccctctagATCTCTCTCTCTCTCTCACACACACAGGG	Cloning REF6 promoter in reporter lines
p3_40	ELF6 promoter	ataattcgagggtacATTGCTCGTTTAACAAGACCGTG	Cloning ELF6 promoter in reporter lines
p3_41	ELF6 promoter	cgtcgggccctctagCTTAAATTCCCAATTCCGTAAGACC	Cloning ELF6 promoter in reporter lines
p3_42	BRL3 promoter	GAGCTCGTTCGGCGCAAATACT CDAC	Cloning BRL3 reporter construct
p3_43	BRL3 promoter	GCTAGCGTTATTAGCCCACAAAGTGTTCGA	Cloning BRL3 reporter construct
